# Muscle Strength and Functional Ability in Recreational Female Golfers and Less Active Non-Golfers over the Age of 80 Years

**DOI:** 10.3390/geriatrics2010012

**Published:** 2017-03-04

**Authors:** Alison Stockdale, Nicholas Webb, Jessica Wootton, Jonathan Drennan, Simon Brown, Maria Stokes

**Affiliations:** 1 Faculty of Health Sciences, Building 45, University of Southampton, Southampton SO17 1BJ, UK; a.stockdale1@nhs.net (A.S.); nicholas.webb4@nhs.net (N.W.); jessica.wootton@gwh.nhs.uk (J.W.); simon.brown@soton.ac.uk (S.B.); 2 School of Nursing & Midwifery, University College Cork, Cork T12 AK54, Ireland; jonathan.drennan@ucc.ie; 3 Arthritis Research UK Centre for Sport, Exercise and Osteoarthritis, Nottingham NG7 2UH, UK

**Keywords:** older females, physical activity, golf, muscle strength, sarcopenia

## Abstract

Muscle strength and functional ability decline with age. Physical activity can slow the decline but whether recreational golf is associated with slower decline is unknown. This cross-sectional, observational study aimed to examine the feasibility of testing muscle strength and functional ability in older female golfers and non-golfers in community settings. Thirty-one females over aged 80, living independently (golfers *n* = 21, mean age 83, standard deviation (±) 2.1 years); non-golfers, *n* = 10 (80.8 ± 1.03 years) were studied. Maximal isometric contractions of handgrip and quadriceps were tested on the dominant side. Functional ability was assessed using the Timed Up and Go (TUG) and health-related quality of life using the Short Form-36 questionnaire. Grip strength, normalised to body mass, was greater in golfers (0.33 ± 0.06 kgF/kg) than non-golfers (0.29 ± 0.06), however, the difference was not statistically significant (*p* = 0.051). Quadriceps strength did not differ (golfers 2.78 ± 0.74 N/kg; non-golfers 2.69 ± 0.83; *p* = 0.774). TUG times were significantly faster (*p* = 0.027) in golfers (10.4 ± 1.9 s) than non-golfers (12.6 ± 3.21 s; within sarcopenic category). Quality of life was significantly higher in golfers for the physical categories (Physical Function *p* < 0.001; Physical *p* = 0.033; Bodily pain *p* = 0.028; Vitality *p* = 0.047) but psychosocial categories did not differ. These findings indicated that the assessment techniques were feasible in both groups and sensitive enough to detect some differences between groups. The indication that golf was associated with better physical function than non-golfers in females over 80 needs to be examined by prospective randomised controlled trials to determine whether golf can help to achieve the recommended guidelines for strengthening exercise to protect against sarcopenia.

## 1. Introduction

By 2037, the number of people aged 80 or over living in the United Kingdom is anticipated to rise to 6 million [1], equating to one in 12 of the population [1]. Progressive declines in muscle strength and functional ability with ageing are well documented, reducing the individual’s independence [2] and leading to sarcopenia [3,4,5]. Exercise, specifically endurance and resistance training, has been shown to improve strength and functional ability, as well as reducing decline of muscle mass [6,7] but older people are not active enough. Of adults aged over 75 years, only 9% of men and 6% of women met the recommended guidelines for physical activity [8].

Strengthening recommendations for older people have been proposed in various guidelines (e.g., American College of Sports Medicine, World Health Organisation, US Department of Health and Human Service, Four Home Nations Chief Medical Officers) [9]. The World Health Organisation recommends that those over 65 years should carry out muscle strengthening activities on two or more days per week [10]. Golf is a popular recreational activity for older people requiring balance, flexibility and stamina, and is known to benefit cardiovascular, musculoskeletal and balance aspects [11,12,13]. However, the potential benefits of golf protecting against sarcopenia are unknown [14].

Grip strength is proposed to be a more effective marker of frailty than age [15,16]. Quadriceps strength directly impacts walking and independence [17], and weakness or asymmetry can be an effective predictor of future falls [18]. The correlation between handgrip and quadriceps strength reduces with age [19], so it may be important to study both muscles in older people. Functional ability can be assessed by a Timed Up and Go (TUG) test [20], which is a simple, rapid test that can be used to identify those at risk of falls in older adults [21]. Quality of life is important to consider in relation to physical and psycho-social well-being and can be assessed using the SF-36 [22].

Older females have the lowest levels of physical activity and are the least researched group in the general population [8]), and are more likely to be diagnosed with sarcopenia [3]. The present cross-sectional study aimed to test the feasibility of using portable tests in a community setting, in terms of convenience and ease of use of equipment (both for investigators and participants) to aid recruitment to community-based research, and whether the techniques used were sensitive enough to detect differences in muscle strength and functional ability between recreational golfers aged over 80 years and those less active, to see if larger prospective studies were warranted.

## 2. Materials and Methods

### 2.1. Participant Recruitment

Females aged 80 years and over were recruited from the local community in Hampshire. Golfers were recruited through golf clubs using posters. Non-golfers were recruited through sheltered housing and the University of Southampton Psychology Register. Ethical approval was obtained from the Faculty of Health Sciences Ethics Committee, University of Southampton (Ethics number: 14158). A screening questionnaire was completed over the telephone to establish eligibility. Prior to testing, all procedures were explained to participants and written informed consent obtained, and the Physical Activity Scale for the Elderly (PASE) questionnaire [23], and a health related quality of life (HRQoL) Short Form-36 questionnaire (SF-36) completed (non-commercial license, Optuminsight Life Sciences Inc., “Optum”, Lincoln, RI, USA). Both questionnaires used, the PASE [23] and SF-36 [22] were selected as they are well established and known to be robust in terms of validity and reliability.

A convenience sample of 31 females was recruited (golfers = 21, non-golfers = 10). Inclusion criteria were: mobile, living independently and cognitively able to understand study requirements. Controlled hypertension or systematic illnesses, such as diabetes, antidepressant use or previous cancers and surgery that did not negatively affect a participant’s function were permitted to reflect the older general population. In those with joint replacements, maximal strength tests were either completed on the non-dominant side or not at all if the individual had bilateral joint replacements. Exclusion criteria were: musculoskeletal injury in the past five years that resulted in immobility for more than one week; a known neurological disorder; arthritis which restricted the ability to perform every day activities; receiving treatment for cancer; taking medication which affects muscle function; upper or lower limb prostheses, spinal surgery, inability to perform painless maximal contractions or active inflammatory joint disease.

Golfers walked the course, played 18 holes at least once per week and had played for two years or more. Three individuals who only played nine holes were included as they played more than once a week and had played golf regularly for at least 10 years. Individuals who completed any regular moderate exercise, as determined by the PASE questionnaire [23], were excluded from the non-golfers group.

### 2.2. Protocol

Body mass and height were measured, and body mass index calculated (BMI). The dominant upper and lower limbs were tested and were determined by writing hand and preferred leg for kicking a football, respectively. Participants performed two sub-maximal contractions with one minute intervals prior to strength testing, to familiarise themselves with the equipment and as a warm up. Both strength tests followed a protocol of three consecutive maximal contractions, lasting three seconds each, and a 30 s rest period between each [19]. The maximal recorded value was used for data analysis. Verbal encouragement was given to participants.

#### 2.2.1. Handgrip Strength

Grip strength was measured using a JAMAR dynamometer (model J00105), with the handle set in the second position. Participants were tested in a seated position, with neutral shoulder adduction and rotation, elbow flexed to 90° and forearm positioned neutrally resting on a support [19]. Previous studies have recorded excellent reliability (intraclass correlation coefficients of 0.97–0.98) in older adults using this method to measure grip strength [24].

#### 2.2.2. Quadriceps Strength

Participants were seated in a high chair on a plinth, with hips and knees at 90° and feet off the floor. An electronic Im-Able Hand-Held Dynamometer (HHD) was positioned on the anterior tibia, just proximal to the malleoli. Isometric force was recorded as the participant attempted to extend their knee.

#### 2.2.3. Timed Up and Go (TUG)

The TUG test followed a standardised protocol [20], which involved recording the time taken for a participant to stand from a chair, walk 3 m and return to being seated on the chair.

### 2.3. Data Analysis

Descriptive statistics were computed in Excel and analysed in SPSS version 16.0 (SPSS Inc. 2007, Chicago, IL, USA). Body mass index (BMI) was calculated (mass divided by height squared). Where data were normally distributed, assessed using the Shapiro-Wilk test, they were expressed as means and standard deviations and when not normally distributed, were expressed as medians and interquartile ranges (IQR). Handgrip and quadriceps strength were expressed relative to body mass by dividing the strength value (kgF for handgrip and Newtons for quadriceps) by body mass (kg). The ratio of participants’ handgrip to quadriceps strength was calculated (HG/Q). Analysis examined differences between golfers and non-golfers, using the independent *t-*test (parametric for normally distributed) or Mann Whitney U Test (non-parametric). Statistical significance was set at *p* < 0.05.

## 3. Results

### 3.1. Demographic Details

Golfers were older than non-golfers; however, there were no other statistically significant differences in participant characteristics between groups (Table 1).

Three non-golfers had non-dominant measurements taken for quadriceps testing (due to hip replacements on the dominant side) and three participants did not complete lower limb testing as they had bilateral hip replacements (golfers = 2, non-golfers = 1).

### 3.2. Physical Function Tests

The results of Shapiro-Wilk tests confirmed normal distribution for the physical tests. Grip strength *per se* did not differ (*p* = 0.190) between golfers (20.6 ± 2.14: range 16–26 KgF) and non-golfers (18.9 ± 4.81: range 13−28 KgF) but when normalised for body mass, grip was greater in golfers (0.33 ± 0.06 kgF/kg) than non-golfers (0.29 ± 0.06); however, the difference was not statistically significant (*p* = 0.051; Table 2). One handgrip measurement was excluded, as an incorrect handle setting was used on the JAMAR dynamometer. Quadriceps strength did not differ between groups, whether for actual values (golfers 170.2 ± 42.9, 103.0–274.7 Newtons; non-golfers 174.4 ± 53.2, 91.2–237.4) or normalised (golfers 2.78 ± 0.74 N/kg; non-golfers 2.69 ± 0.83; Table 2). Quadriceps testing was completed on the non-dominant side in those with a lower limb joint replacement and omitted in those with bilateral joint replacements, hence the varying numbers of participants indicated in Table 2. Golfers’ TUG times (10.4 ± 1.9 s) were significantly faster (*p* = 0.027) than non-golfers’ (12.6 ± 3.21; Table 2).

### 3.3. Quality of Life

**Figure 1:**
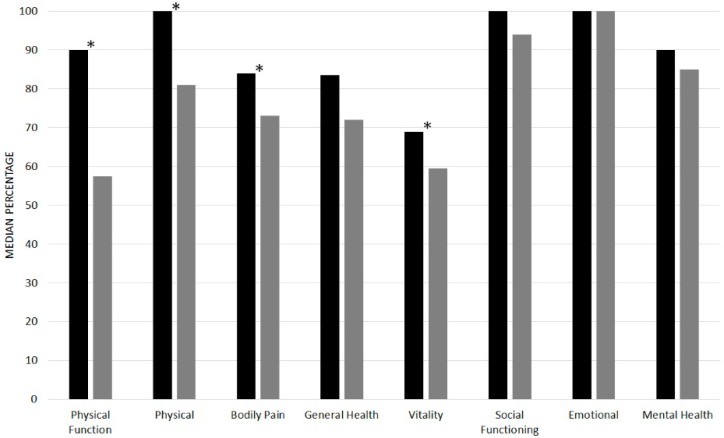
Quality of life Short Form (SF)-36 category scores for golfers and non-golfers, where 100% represents no disability and 0% indicates the worst possible level of functioning. * Significant difference between 2 groups (*p* < 0.05).

Quality of life scores (SF-36) were significantly higher in golfers than non-golfers for the physical categories (Physical Function *p* < 0.001, Physical *p* = 0.033, Bodily pain *p* = 0.028 and Vitality *p* = 0.047) but there were no significant differences in the psychosocial categories (Figure 1).

## 4. Discussion

The present study has shown that the methods used to assess function and quality of life were feasible for testing females over the age of 80 years in a community setting. Better TUG scores could be detected in golfers than non-golfers, despite no differences in quadriceps strength on the (dominant) side tested. An important novel finding was that golfers’ TUG scores were above the threshold for predicting sarcopenia [25] but the non-golfers were classified as being sarcopenic. In the study that determined the TUG threshold, the diagnostic criteria for sarcopenia were a reduction in skeletal muscle mass and handgrip strength, and/or poor physical performance in a 6-m gait-speed test [25]. Although these preliminary findings suggest that golf may be associated with training effects that fulfil the recommendations for older people [10] the study design did not allow such conclusions to be drawn and this needs to be examined in prospective randomised controlled trials of those new to golf [14].

The golf swing technique may have a strength training effect on grip strength, as grip was greater on the non-dominant side in professional male golfers, probably due to the nature of the golf grip and swing technique [26]. Bilateral testing was not performed in the present study and it has yet to be determined if asymmetry applies to recreational golfers over the age spectrum. Several other factors may influence differences in grip strength between golfers and non-golfers, such as lifting and carrying of clubs, or that individuals who recreationally play golf and walk the course might have better general health [27].

A recent cross-sectional study analysed the accuracy of TUG to predict sarcopenia and found the cut-off point longer than or equal to 10.85 seconds [25]; the data being generated from a mixed gender, hospital inpatient population (70.4 ± 7.7 years). In the present study, non-golfers had TUG scores below that threshold (mean 12.6 s) but the golfers did not (despite being older than their control group), which may imply that recreational golf can help prevent sarcopenia but the present study only demonstrated an association between golf and better TUG scores; a prospective study is needed to determine the training effects of golf. The golfers were also functionally more able, according to the self-rated SF-36 self-assessment scores.

The similarity in isometric quadriceps strength between groups indicated that recreational golf, walking the course might not be associated with a specific effect on quadriceps on the dominant side in older females over 80. The non-dominant side was not studied but may have shown a difference, as this is the leading leg and bears greater load during the swing through. The quadriceps muscle is needed for speed and power, the ability to produce maximal force in a short period of time, which are characteristic of type 2 fibres and decline faster than type 1 fibres with ageing [18,28,29]. Isometric testing using the portable dynamometer was more feasible than using isokinetic dynamometry in this community-based study, so differences in muscle power may have been missed. The differences found in functional testing suggest that this was the case, as rising from a chair during the TUG test required muscle power and may have shown a correlation with muscle power, had the latter been measured.

The small sample size was a limitation of the present study and may have accounted for difference in handgrip. The cross-sectional study design comparing golfers, who were self-selected, with sedentary controls cannot be used to determine the training effects of golf, which needs to be examined in a randomised controlled prospective trial but the present findings indicate that such a study is warranted. Future studies will need to minimise potential differences, other than golf, that may exist between groups that were not controlled for in the present study and may have contributed to the findings. These factors include: using a recruitment strategy to capture golfers and non-golfers from the same environments; accounting for other physical activities undertaken and levels of activity; and ensuring similar socio-economic status.

Studying the dominant side only was another limitation, as the specific training effects of golf would be expected to occur on the non-dominant side for reasons explained above. The dominant side was studied, as it is usually tested to represent general strength. Also, the present study protocol needed to limit the number of tests to ensure data collection sessions, which included other related studies addressing different research questions using different technologies, did not over burden participants and minimised the potential for fatigue. Future studies could include bilateral measurements.

Testing quadriceps power may have provided a more relevant indication of functional capacity but isometric testing was more feasible for a field situation out in the community. Quadriceps strength was tested with the participant seated upright, as hip and knee joint angles are easy to standardise at 90 degrees and replicate in this position. However, the contraction was difficult to oppose manually in some of the stronger participants, which could potentially have resulted in an underestimation of their maximal force. Testing in supine, with a knee supported at a low angle (e.g., 20 degrees) measuring knee extension in outer range [30], may be a more appropriate position for future studies. Another way of assessing muscle is to measure its size, as this is known to be closely related to strength [31]. A related study measured quadriceps thickness using ultrasound imaging in females golfers (*n* = 31) and non-golfers (*n* = 35) aged 65–79 years, and found greater muscle thickness in golfers [32]. As in the present study, feasibility of using ultrasound imaging was demonstrated and differences between groups showed the technique was sensitive for distinguishing between groups.

The present study was feasible in a community setting in the older females studied, although costs, participant burden etc. were not measured to compare laboratory and field based testing more fully. Anecdotal evidence indicates that the mobile nature of the study enhanced recruitment, as some participants commented they would not have been willing to travel to the university and some said they would not like to be tested in a laboratory. The procedures were acceptable and relatively easy to perform, and all participants completed the tests required of them. Future studies could include an economic evaluation of field testing and take steps to optimise researchers’ time spent travelling, e.g., by scheduling a minimum number of participants for each session. Recruitment of control participants was more challenging than golfers, so future studies will require robust recruitment strategies, perhaps including the media.

Although physical function would be expected to be better in older females who play golf than those who are less active, it is still important to conduct prospective studies to provide objective evidence of this difference to determine the specific effects of various activities to see if they help to achieve the recommended guidelines for exercise to protect against sarcopenia. Such evidence is needed to help influence policy makers to broaden access to specific activities, such as golf.

## 5. Conclusions

The physical tests used were feasible for use in the community environment and modifications have been proposed. Recreational golf in females aged over 80 appeared to be associated with significantly better physical function, in terms of TUG and physical outcomes as measured by the SF-36, than non-golfers. Grip strength was greater in golfers; however, the difference between cohorts was not statistically significant; in addition, isometric quadriceps’ strength did not differ between groups. Prospective studies are warranted to study the training effects and determine whether golf can help protect against sarcopenia.

## Figures and Tables

**Table 1:** Participant characteristics of study groups.

Characteristic	Golfer (*n* = 21)	Non-Golfer (*n* = 10)
Age (years)	82.9 ± 2.1 (80–87)	80.8 * ± 1.03 (80–83)
Height (m)	1.59 ± 0.05 (1.51–1.65)	1.57 ± 0.06 (1.47–1.67)
Weight (kg)	62.3 ± 9.22 (46.9–78.8)	66.2 ± 12.76 (53.4–93.8)
BMI (kg/cm²)	24.7 ± 3.55 (18.78–31.93)	26.77 ± 4.4 (20.78–33.84)

* Significantly different between 2 groups (*p* < 0.05).

**Table 2:** Physical function results: handgrip and quadriceps strength (relative to body mass), handgrip and quadriceps ratio and Timed Up and Go (TUG).

Function	Golfer	Non-Golfer	*p* Value
Handgrip/BW (kgF/kg)	0.33 ± 0.06 (0.24–0.46) (*n* = 20)	0.29 ± 0.06 (0.24–0.39) (*n* = 9)	0.051
Quads/BW (N/kg)	2.78 ± 0.74 (1.6–4.4) (*n* = 19)	2.69 ± 0.83 (1.71–4.00) (*n* = 8)	0.774
HG/Q ratio	1.27 ± 0.35 (0.79–2.00) (*n* = 18)	1.17 ± 0.51 (0.58–2.26) (*n* = 8)	0.565
* TUG (s)	10.4 ± 1.95 (7.3–14.9) (*n* = 21)	12.6 ± 3.21 (8.9–18.1) (*n* = 10)	* 0.027

Abbreviations: /BW = relative to body weight. HG/Q = ratio of handgrip/quadriceps strength. Data are mean, standard deviation and range. Missing data are indicated by (*n* = *x*) after the range. * Statistically significant difference between groups.
